# Optimising a method for aragonite precipitation in simulated biogenic calcification media

**DOI:** 10.1371/journal.pone.0278627

**Published:** 2022-12-02

**Authors:** Celeste Kellock, Maria Cristina Castillo Alvarez, Adrian Finch, Kirsty Penkman, Roland Kröger, Matthieu Clog, Nicola Allison

**Affiliations:** 1 School of Earth and Environmental Sciences, University of St. Andrews, St. Andrews, United Kingdom; 2 Department of Chemistry, University of York, York, United Kingdom; 3 Department of Physics, University of York, York, United Kingdom; 4 Scottish Universities Environmental Research Centre, Glasgow, United Kingdom; VIT University, INDIA

## Abstract

Resolving how factors such as temperature, pH, biomolecules and mineral growth rate influence the geochemistry and structure of biogenic CaCO_3_, is essential to the effective development of palaeoproxies. Here we optimise a method to precipitate the CaCO_3_ polymorph aragonite from seawater, under tightly controlled conditions that simulate the saturation state (Ω) of coral calcification fluids. We then use the method to explore the influence of aspartic acid (one of the most abundant amino acids in coral skeletons) on aragonite structure and morphology. Using ≥200 mg of aragonite seed (surface area 0.84 m^2^), to provide a surface for mineral growth, in a 330 mL seawater volume, generates reproducible estimates of precipitation rate over Ω_aragonite_ = 6.9–19.2. However, unseeded precipitations are highly variable in duration and do not provide consistent estimates of precipitation rate. Low concentrations of aspartic acid (1–10 μM) promote aragonite formation, but high concentrations (≥ 1 mM) inhibit precipitation. The Raman spectra of aragonite precipitated *in vitro* can be separated from the signature of the starting seed by ensuring that at least 60% of the analysed aragonite is precipitated *in vitro* (equivalent to using a seed of 200 mg and precipitating 300 mg aragonite *in vitro*). Aspartic acid concentrations ≥ 1mM caused a significant increase in the full width half maxima of the Raman aragonite v_1_ peak, reflective of increased rotational disorder in the aragonite structure. Changes in the organic content of coral skeletons can drive variations in the FWHM of the Raman aragonite ν_1_ peak, and if not accounted for, may confuse the interpretation of calcification fluid saturation state from this parameter.

## 1. Introduction

Marine biogenic carbonates are invaluable archives of past climate information, potentially recording information on seawater composition, temperature and pH in their geochemistry and structure (e.g. the Sr/Ca ratio of aragonite coral skeletons and the Mg/Ca ratio of calcite foraminifera tests are influenced by seawater temperatures) [1, 2]. Rotational disorder in the CaCO_3_ structure is inferred to reflect changes in the dissolved inorganic carbon (DIC) chemistry of the media used for biomineralisation in biogenic carbonates, and may reflect changes in ocean carbonate saturation state [3, 4]. Carbonate proxies are instrumental in shaping our understanding of past climate and critical in validating global climate models for predicting 21st century climate change [5]. In spite of this potential, the relationships between environment, CaCO_3_ geochemistry and structure are poorly understood. CaCO_3_ geochemistry and structural order can be affected by temperature [6, 7], pH and [DIC] [8–10], the presence of organic ligands [11] (which occur in biogenic carbonates) and CaCO_3_ precipitation rate [12, 13]. Resolving how these multiple factors influence CaCO_3_ geochemistry and structure, separately and in combination, is essential to the effective development of palaeoproxies.

**Fig 1:**
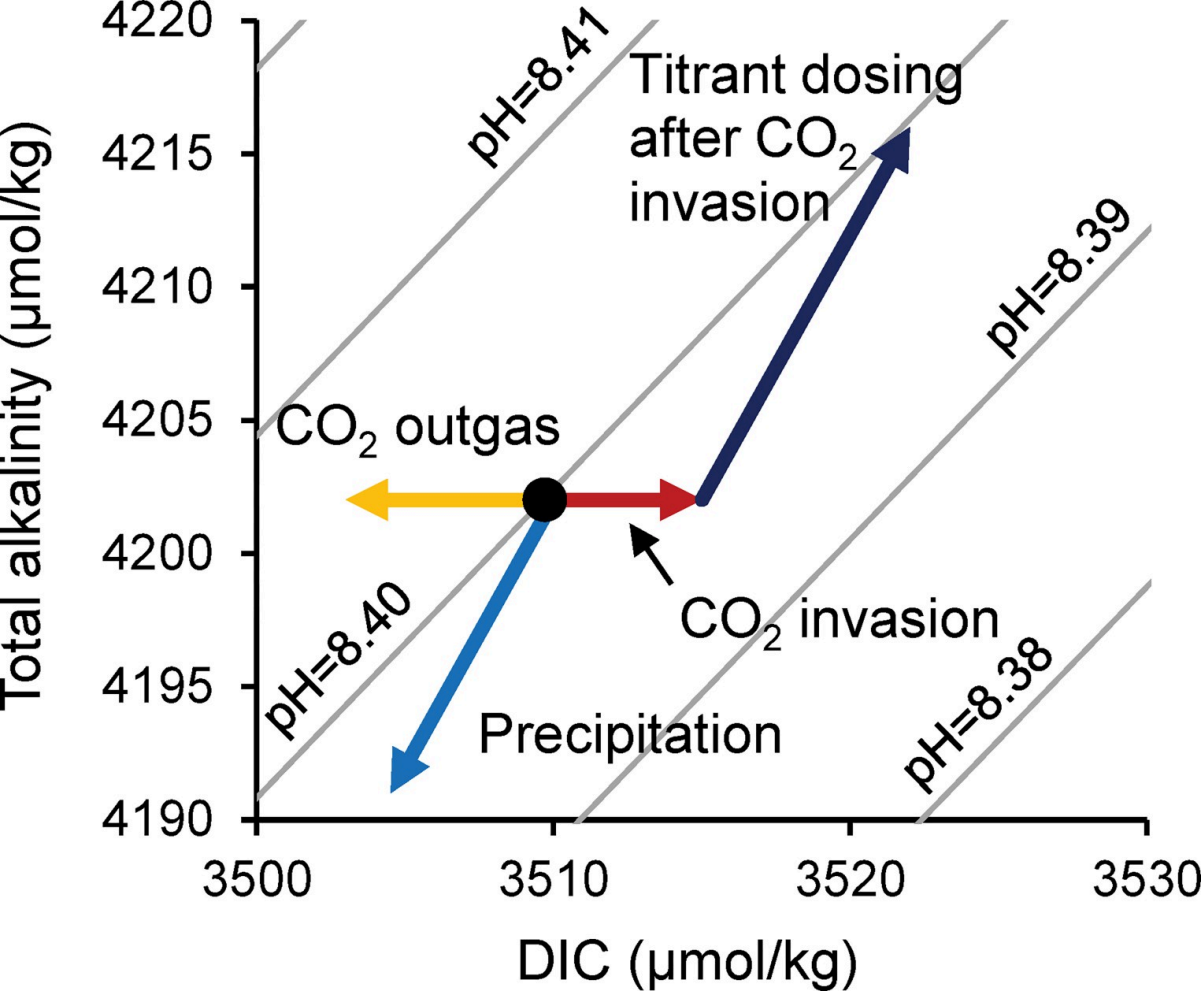
Effect of processes on the DIC chemistry of seawater. Precipitation reduces DIC, alkalinity and hence pH. Titrant dosing after precipitation returns the DIC to the starting composition (black dot). CO_2_ invasion and outgas modify the DIC but not the alkalinity of the seawater. CO_2_ invasion reduces pH and triggers the dosing of titrants which return the seawater to the starting pH but at a different DIC and alkalinity composition. Contours join points of equal pH (NBS scale).

The influence of temperature and solution chemistry on CaCO_3_ geochemistry and structure can be explored in CaCO_3_ precipitations *in vitro*. A range of methods have been adopted for this, including Na_2_CO_3_ addition [6, 9], CO_2_ degassing from high pCO_2_ (1 atm) solutions, pH-stat [9] and ammonium carbonate decomposition [12, 14]. These studies yield many useful insights into the controls on CaCO_3_ chemistry. However, designing experiments which permit CaCO_3_ precipitation under steady state solution conditions that are comparable to those of calcareous organism calcification sites (where the CaCO_3_ mineral is formed) is challenging. A pH-stat can be used to maintain constant solution pH, but significant invasion (or outgassing) of CO_2_ occurs if the solution and surrounding atmosphere are not at equilibrium and this alters the solution DIC chemistry (Fig 1. For example, [DIC] more than doubled when we precipitated aragonite by maintaining a seawater solution at pH 8.7 in an open laboratory. In some reported studies the Ca^2+^ consumed during CaCO_3_ precipitation was not replaced and solution [Ca^2+^] decreased by >70% [9] during experiments. These variations in solution pH, [DIC] and [Ca^2+^] generate large changes in solution CaCO_3_ saturation state (Ω) during the lifetime of the experiment, which in turn affect CaCO_3_ growth rates [15] and may influence geochemistry and structure.

Here we report a study to optimise a method to precipitate aragonite under tightly controlled temperature, pH, Ω, [DIC] and [Ca^2+^] conditions, similar to those of coral calcification media. We conducted precipitations both without and with a seed to provide a nucleation surface for the growth of aragonite *in vitro* and to permit the normalisation of precipitation rate to seed surface area [15]. We investigated the optimal seed surface area in our apparatus to provide consistent results and tested the dynamics of precipitations in both artificial and natural seawaters. We analysed precipitated aragonites using Raman spectroscopy to test how variations in the proportion of seed versus aragonite precipitated *in vitro* affect structure. Raman spectroscopy measures the change in photon frequency (Raman shift) as monochromatic light is scattered inelastically by interaction with molecular vibrations within a material. It is used to identify CaCO_3_ polymorph [16], detect disorder in the CaCO_3_ lattice [3, 4, 16], explore the distribution of organic materials [16] and infer Mg content in biogenic carbonates [3, 4]. Finally, we demonstrate use of our optimised methodology to test the effect of varying the concentration of aspartic acid on aragonite precipitation rate and structure. Aspartic acid is the most common amino acid in Scleractinia coral skeletons [17, 18] as a key component of biomineralisation proteins [19]. Resolving the role that biomolecules play in aragonite precipitation and structure is critical to a full understanding of the biomineralisation process and how this may change under different climate conditions.

## 2. Methods

### 2.1 Precipitation apparatus

To address the aims of the present study, we created an automated precipitation apparatus in which the chemical parameters are maintained at constant values throughout the experiment. We precipitated aragonite from seawater solutions with pH and [DIC] altered from typical seawater values. Precipitation of CaCO_3_ consumes DIC and Ca^2+^ and reduces solution pH (Fig 1). The pH of the solution was constantly monitored using a high precision pH/temperature sensor (Metrohm Aquatrode Pt1000) and a pH decrease triggered an adapted Metrohm Titrando 902 titrator to add equal volumes of 0.6 M Na_2_CO_3_ and CaCl_2_ titrants to replace the ions precipitated from solution. The CaCl_2_ titrant was prepared as 0.594 M CaCl_2_ + 0.006 M SrCl_2_ to ensure replacement of both the Ca and the Sr which can substitute for Ca in the aragonite lattice [20].

For each experiment 330–340 mL of filtered seawater (0.2 μm polyether sulfone filter) was measured into a high-density polyethylene (HDPE) plastic beaker (total volume ~360 mL) and capped with an ethylene tetrafluoroethylene lid with multiple ports. A pH sensor, a propeller stirrer, a gas tube and the 2 titrant dosing tubes were inserted through the lid and into the headspace (gas tube) or seawater (all others). The HDPE beaker was maintained at 25°C by immersion in a heated circulating water bath (Optima TC120) fitted with a cooling coil. To avoid invasion or outgassing of CO_2_ which alter seawater pH (Fig 1) and DIC speciation, the precipitating solution was maintained under a headspace with a filtered ambient air gas stream (pCO_2_ = ~416 μatm) and the pH and DIC of the seawater were adjusted to be in equilibrium with this atmosphere. At the start of each experiment seawater [DIC] was increased with the addition of 0.6 M Na_2_CO_3_ and the pH adjusted to the required value by addition of 1.0 M HCl or NaOH. Once pH was stable the dry, weighed aragonite seed (if used) was suspended in 1 mL of the seawater solution before being added to the reaction vessel. The titration usually proceeded until 5 mL of each titrant were added to the reaction vessel resulting in the precipitation of ~300 mg of aragonite. The pH sensor was calibrated each week with fresh buffers. Over the course of the week the pH of the buffers changed by <0.003 pH units. At the end of each experiment the sensor, beaker and propeller stirrer were submerged in 0.1 M HCl to dissolve any precipitate and then rinsed thoroughly with deionized water. The sensor was acclimated in seawater for at least 30 minutes before reuse.

### 2.2 Testing the effects of Ω and seed mass on aragonite precipitation

Experiments were conducted over a range of Ω and in either artificial or natural seawater (Table 1). Natural seawater was collected from the shore in Crail, Fife, UK, filtered and stored in a blacked-out 1000 L HDPE container for several weeks before use. Artificial seawater was made according to reference [21]. Both waters were bubbled with atmospheric air sourced from outside the building ([CO_2_] ≈ 410–420 ppm) before use. The total alkalinity and DIC (after bubbling) were determined by automated Gran titration (Metrohm, 888 Titrando) and using an Apollo SciTech (AS-C3) DIC Analyser [22]. Both instruments were calibrated with CRM (certified reference material) (A. Dickson, Scripps Institution of Oceanography) and yielded precision (standard deviation) of multiple analyses of <0.2% in each case. Salinity was estimated from conductivity measured with a Thermo Orion 5 star pH/RDO/conductivity meter calibrated with NIST standards.

**Table 1:** Chemistry of the waters used for precipitations. [Ca^*2+*^] and [Mg^*2+*^] is estimated for artificial seawater (based upon composition) and measured (by ICP-OES) for natural seawater.

	Natural seawater	Artificial seawater
Total alkalinity (μmol kg^-1^)	2208	2309
DIC (μmol/kg^-1^) when bubbled at ambient pCO_2_	1933	2015
Salinity	34.1	33.7
[Ca^2+^] mM	10.1	9.9
[Mg^2+^] mM	51	53

Precipitations were either conducted using no seed or using 50, 100, 200 or 400 mg of an aragonite seed produced by wet grinding pieces of a *Porites lutea* coral skeleton in an agate ball mill. The seed had a surface area of 4.2 m^2^ g^-1^ determined by the Brunauer-Emmett-Teller technique, assuming a density of aragonite of 2.94 g cm^-3^. Ω_aragonite_ (hereafter abbreviated to Ω) were calculated using [Ca^2+^] (Table 1) and [CO_3_^2-^] and the solubility product (K_sp_) of aragonite at 25°C and 1 atmosphere [23]. Seawater [CO_3_^2-^] is calculated using CO2 sys v2.1 [reference 24] from seawater [DIC] and pH_NBS_ using the equilibrium constants for carbonic acid and KHSO_4_ [references 25, 26] and total [B] [reference 27]. The small variation in [Ca^2+^] between the artificial and natural seawater altered Ω by ≤0.2 and we consider this insignificant in terms of the Ω range studied. Experiments were conducted at a pH_NBS_ = 8.337 with DIC = 3000 μmol kg^-1^, pH_NBS_ = 8.445 with DIC = 4000 μmol kg^-1^ and pH_NBS_ = 8.564 with DIC = 5500 μmol kg^-1^. Mean Ω of the natural and artificial seawaters at each pH were 6.9, 11.3 and 19.2 respectively. Ω of coral calcification media is estimated to be ~12 based on microsensor measurements of media pH and [CO_3_^2-^] [reference 28].

Using this methodology, pH variations within a precipitation were <0.002 pH units (1σ). Seawater temperatures within and between precipitations varied by <0.3°C. To confirm the seawater conditions, [DIC] was measured at varying time points in a subset of precipitations after filtering the seawater through a 0.2 μm polyethersulfone filter.

The experiment durations were long for unseeded experiments (up to 32 hours) and these were only conducted at Ω = 6.9 and 19.9 in artificial seawater. Durations were also long for experiments using 50 mg of seed at Ω = 6.9 (up to 12 hours) and these titrations were stopped after dosing of 2 mL of each titrant to prevent CO_2_ invasion. Other seeded experiments took from 13 minutes to ~8 hours.

### 2.3 Testing the effect of seed on Raman spectroscopy signature of *in vitro* precipitate

We conducted multiple precipitation experiments at Ω = 11.3 (pH_NBS_ = 8.445, DIC = 4000 μmol kg^-1^) in artificial seawater using 200 mg of the aragonite seed and varying the volume of each titrant dosed into the solution from 1.67 mL to 13.3 mL. Changing the titrant volume resulted in the amount of aragonite precipitated *in vitro* varying between 100 and 800 mg. At the end of each experiment, the CaCO_3_ in the reaction vessel (a mixture of the original seed and the experimental precipitate) was collected by filtration onto a 0.2 μm polycarbonate track etched membrane filters (Cytiva Whatman), rinsed with deionised water and dried at 40°C. Raman spectra of the precipitates and the original seed were collected between 100–1311 wave numbers with a Renishaw In-Via Qontor Raman Microscope using a NIR 300 mW 785 nm solid state laser with a 1200 cm^-1^ grating. We use Raman data to confirm the CaCO_3_ polymorph formed and to explore the degree of internal rotational disorder. Here we focus on the ν_1_ peak, typically the highest intensity peak in the aragonite spectrum. The laser was focused to ~10 x 1 μm and positioned lengthwise along the edges of particles i.e. where *in vitro* precipitation occurs. The instrument was calibrated by measuring an internal Si standard. Multiple spectra (n = 10 or 11) were collected from each sample using a 5% laser power and summing 10 scans with a total acquisition time of 20 s. The spectra were processed using OriginLabs software and the full width half maxima (FWHM) of the *v*_1_ peak were estimated following a Voigt fit which typically yields a better coefficient of determinations than a Gaussian fit and provides a better model of individual vibration bands combined with instrumental artefacts [29]. We tested for variations in the peak position and FWHM between samples using one way ANOVA followed by Tukey’s pairwise comparison.

### 2.4 Testing the effect of aspartic acid on precipitation rate and Raman signature

To test our optimised method we conducted aragonite precipitations in artificial seawater at [aspartic acid] from 1 μM to 8.9 mM. All experiments were conducted at pH_NBS_ = 8.445, [DIC] = 4000 μmol kg ^-1^. Ω = 11.2 and using a seed mass of 200 mg. The aspartic acid (if used) was added to the precipitation seawater before altering the DIC by either suspending the mass of amino acid (L-aspartic acid, Sigma Life Science, >98% purity) required to obtain the final seawater concentration in 1 mL of seawater and then pipetting this into the reaction vessel (for 1 and 8.7 mM) or by dissolving a known mass of amino acid in seawater to produce a stock solution and pipetting aliquots of this into the reaction vessel (for ≤100 μM). Stock aspartic acid solutions were used within 2 hours and then discarded. Precipitates were collected by filtration, dried and characterised by Raman spectroscopy as before. We tested for variations in the peak centre and FWHM of the aragonites using one way ANOVA followed by Tukey’s pairwise comparison.

### 2.5 Scanning electron microscopy

Precipitates were examined by scanning electron microscopy using a JEOL 7800F using a 3kV accelerating voltage at the University of York. Samples were mounted on carbon tabs and no coating was applied.

## 3. Results and discussion

### 3.1 Precipitate characterisation

**Fig 2:**
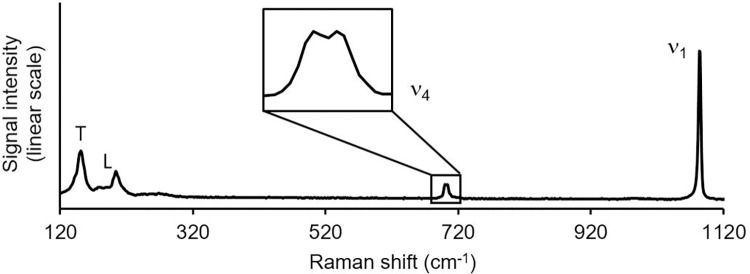
Example Raman spectrum. The material is identified as CaCO_3_ based on the strong ν_1_ peak at ~1084 cm^-1^ and as aragonite based on the dual peak (v_4_) between 700–710 cm^-1^ [reference 30].

Raman spectroscopy (Fig 2) (conducted on at least one precipitate produced under each set of conditions) confirmed that the original seed and precipitates were aragonite. Aragonite has lower symmetry than calcite and hence a single peak in rhombohedral calcite appears as characteristic doublets in orthorhombic aragonite between 700–710 cm-1 [30]. The Raman spectrum of solid aspartic acid indicates that the amino acid has multiple peaks between 120–1120 cm^-1^ including one that coincides with the aragonite ν1 peak (S1 Fig). However, the large aspartic acid peak at ~936 cm^-1^ was never observed in the aragonite spectra and we conclude that any contribution of aspartic acid to the aragonite ν1 peak is insignificant.

### 3.2 Precipitation in unseeded experiments

**Fig 3:**
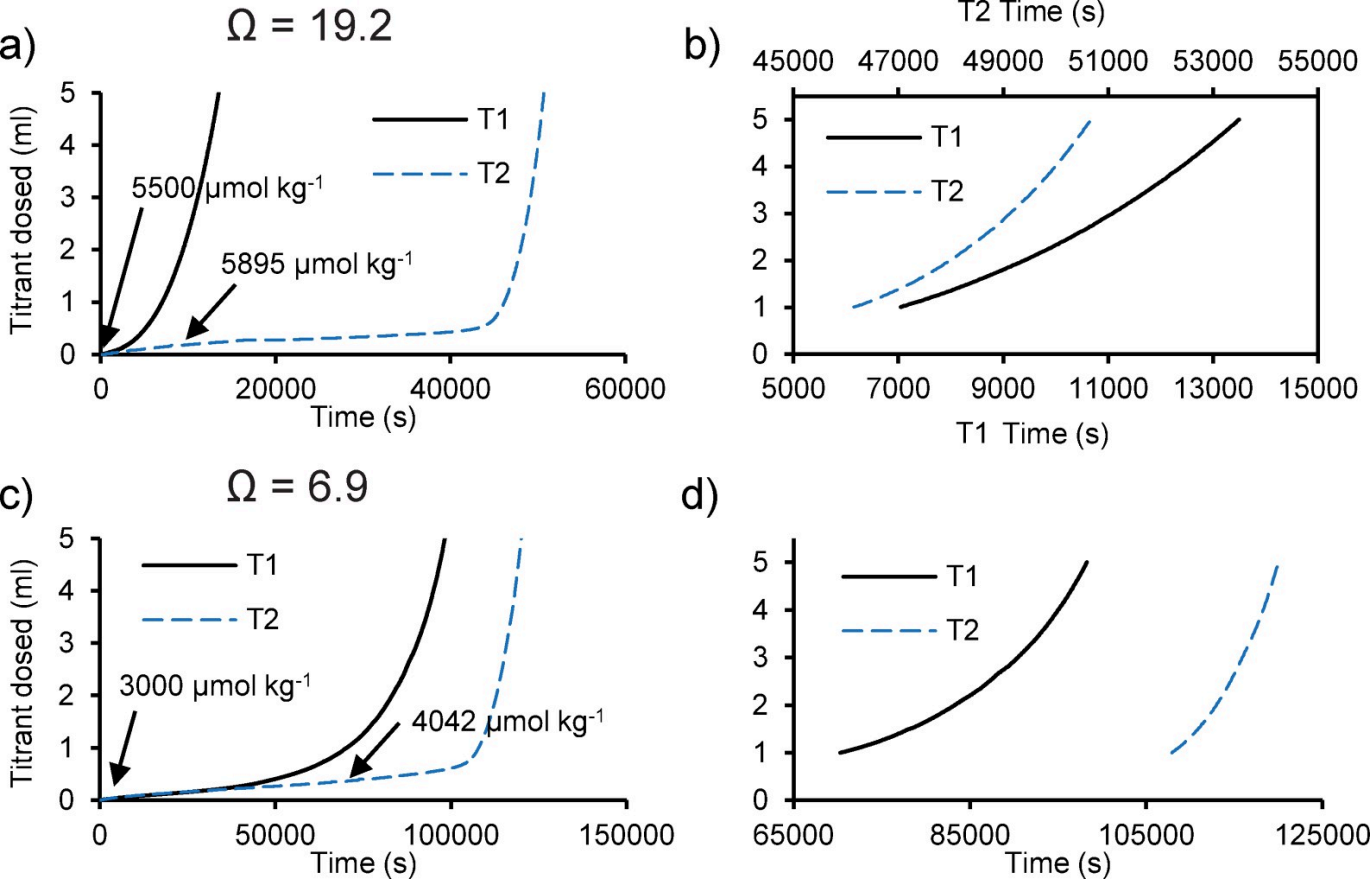
Profiles of titrant volume dosed over time in duplicate unseeded experiments in artificial seawater. a), b) Ω = 19.2 and c), d) Ω = 6.9. b) and d) showed expanded x axes to compare rates of dosing of 1–5 mLs of titrant between duplicates (T1 and T2, show data from two titrators). Measured [DIC] at the start and during 2 of the precipitations are overlaid onto the graphs.

We observed precipitation of aragonite in unseeded experiments in artificial seawater at both Ω = 6.9 and 19.2 (Fig 3). In some of these experiments the titration profile follows a smooth curve and the dosing rate accelerated as the precipitation proceeded (e.g. T1 Fig 3A and 3C). In other experiments (e.g. Fig 3A and 3C, T2) there was a period of relatively little dosing (a lag period) followed by increasingly rapid titrant addition. In one experiment, this lag period exceeds 28 hours (Fig 3). We measured the [DIC] at the start and at a midpoint in the lag of 2 of the titrations (Fig 3C) and observed an increase in DIC between these points. To identify the source of this DIC increase we used CO_2_ sys to estimate the total alkalinity of the seawater at the start and the lag midway point from the measured solution pH_NBS_ and [DIC]. The total alkalinity increased by 1186 and 480 μmol kg^-1^ at Ω = 6.9 and 19.2 respectively. At these midway points the titrator had dosed 0.318 and 0.176 mL of 0.6 M Na_2_CO_3_ respectively, sufficient to increase the total alkalinity of a 330 mL volume by 1156 and 640 μmol kg^-1^. The good agreement between the observed alkalinity increases and that predicted to occur due to the titrant dosing suggests that little CaCO_3_ precipitation occurs during the lag period. [DIC] increased by 1042 and 395 μmol kg^-1^ at the midway lag point in Ω = 6.9 and 19.2 respectively, although titrant dosing is predicted to increase [DIC] by 578 and 320 μmol kg^-1^. The observed increase in [DIC] above that predicted from titrant dosing indicates an invasion of atmospheric CO_2_ from the atmosphere into the seawater solution, i.e. due to a minor disequilibrium. This invasion reduces the solution pH and leads to the dosing of titrant, even in the absence of aragonite precipitation. The increase in solution [DIC] due to CO_2_ invasion is 26 and 31 μmol kg^-1^ h^-1^ at Ω = 6.9 and 19.2 respectively.

Precipitation in the absence of an existing solid surface is termed homogenous nucleation and proceeds as constituent ions of the solid combine to form pre-nucleation clusters which aggregate and dehydrate to form amorphous nanoparticles that ultimately transform into crystals [31]. Homogeneous nucleation of CaCO_3_ is not observed in seawater at 25°C and below Ω_aragonite_ ≈ 12 (i.e. below Ω_calcite_ = 18, [references 32, 33]). As minor CO_2_ invasion into the seawater reduces solution pH and triggers titrant dosing, nucleation may occur in the vicinity of the titrant dosing tube if a higher Ω is achieved at this location. Once CaCO_3_ has formed, this acts as a nucleation surface for subsequent growth. We observe large variations in titrant dosing rate between duplicate experiments, suggesting that the size of the precipitating CaCO_3_ surface is inconsistent between experiments. This hampers the accurate calculation of a precipitation rate normalised to surface area which is required to investigate the influence of crystal growth rate on geochemistry and structure.

### 3.3 Precipitation in seeded experiments

#### 3.3.1. Effect of seed mass

**Fig 4:**
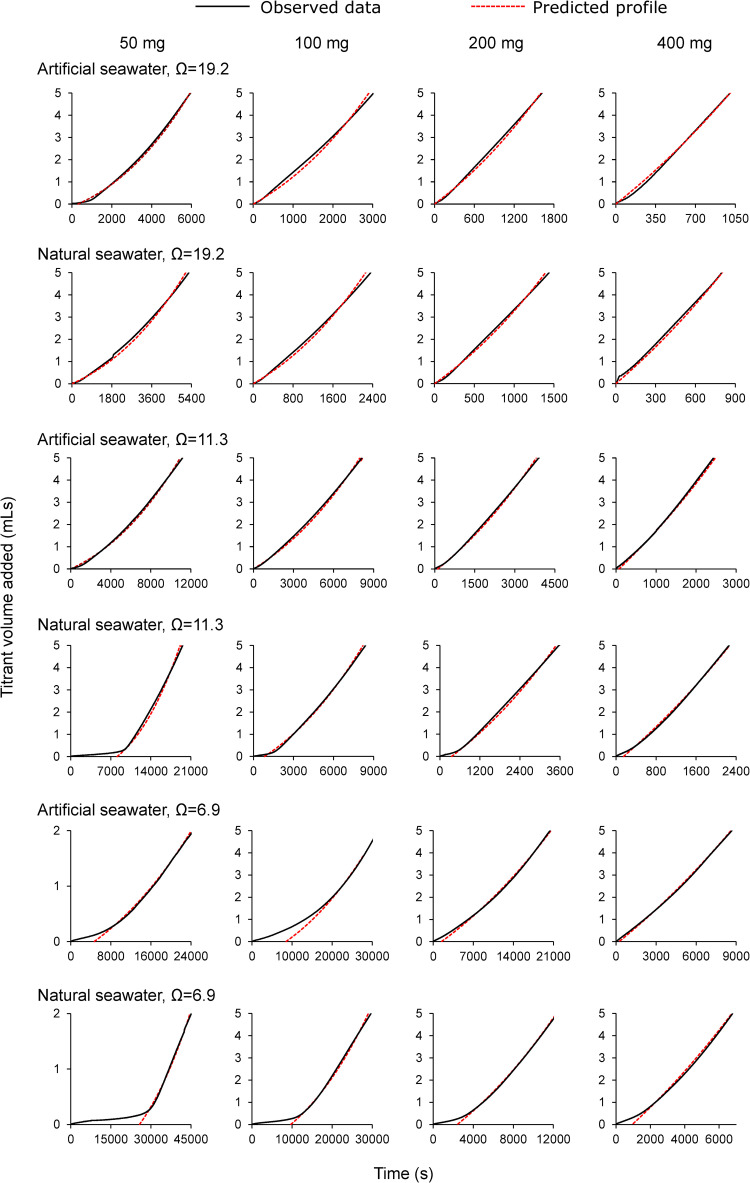
Titration profiles in seeded experiments with no added aspartic acid. Black lines show observed profile and red dotted lines show predicted profile as explained in text. N.B. the x- and y-axes are not to the same scale for all the graphs.

**Fig 5:**
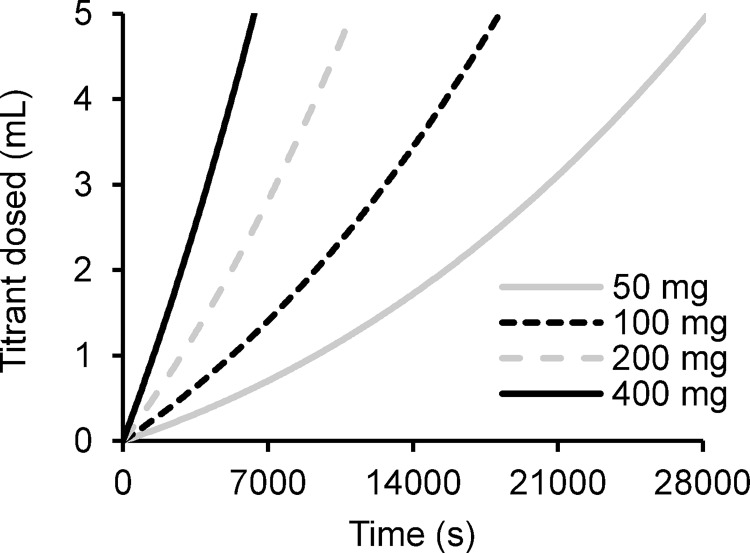
Predicted titrant dosing profiles assuming that all dosing replaces ions consumed in the epitaxial growth of CaCO_3_ over the seed. In this example we assume a precipitation rate of 617 μmol m^-2^ h^-1^ onto a seed of surface area of 4.2 m^2^ g^-1^ using different masses of seed and 0.6 M titrants.

Aragonite was precipitated in all seeded experiments (Fig 4). In some precipitations the rate of titrant dosing was approximately constant resulting in a linear relationship between time and the volume of titrant dosed, while in others the rate of titrant addition increased during the experiment resulting in a curved profile. To explore the origin of these profiles we calculated the profile expected for a series of seeded precipitations assuming that precipitation occurs by epitaxial growth creating a layer all over the starting seed. We selected a precipitation rate of 617 μmol m^-2^ h^-1^ (as for Ω = 6.9 in natural seawater [18]), a seed surface area of 4.2 m^2^ g^-1^, a total dosing of 5 mLs of 0.6 M titrants and masses of starting seed of 400, 200, 100 and 50 mg. We worked with the following approximations: (i) the starting seed is cubic with equal dimensions of 0.486 μm (equivalent to a surface area of 4.2 m^2^ g^-1^ assuming an aragonite density of 2.94 g cm^-3^) and (ii) precipitation occurs by epitaxial growth all over the cubes. We estimate the total surface area of the seed at the start of the experiment, calculate the precipitation that will occur over a set period (typically 10–60 s depending on the amount of seed used) and estimate the volume of titrant required to replace the ions consumed in precipitation. We recalculate the surface area of the precipitate at the end of this time period and continue with these estimates until the end of the precipitation (the time point at which 5 mL of 0.6 M titrant has dosed). While it is unlikely that precipitation occurs in such an idealised manner, our approach explores if the surface area available for precipitation during the titration increases by a similar magnitude to that predicted from epitaxial growth. At higher seed mass the predicted titration profile is approximately linear (Fig 5) as precipitation has little effect on the total surface area available for aragonite growth and titrant dosing rate remains approximately constant. At low seed mass, precipitation significantly increases the surface area available for precipitation as the experiment proceeds and the rate of titrant addition accelerates during the titration making the profile more curved (Fig 5).

**Fig 6:**
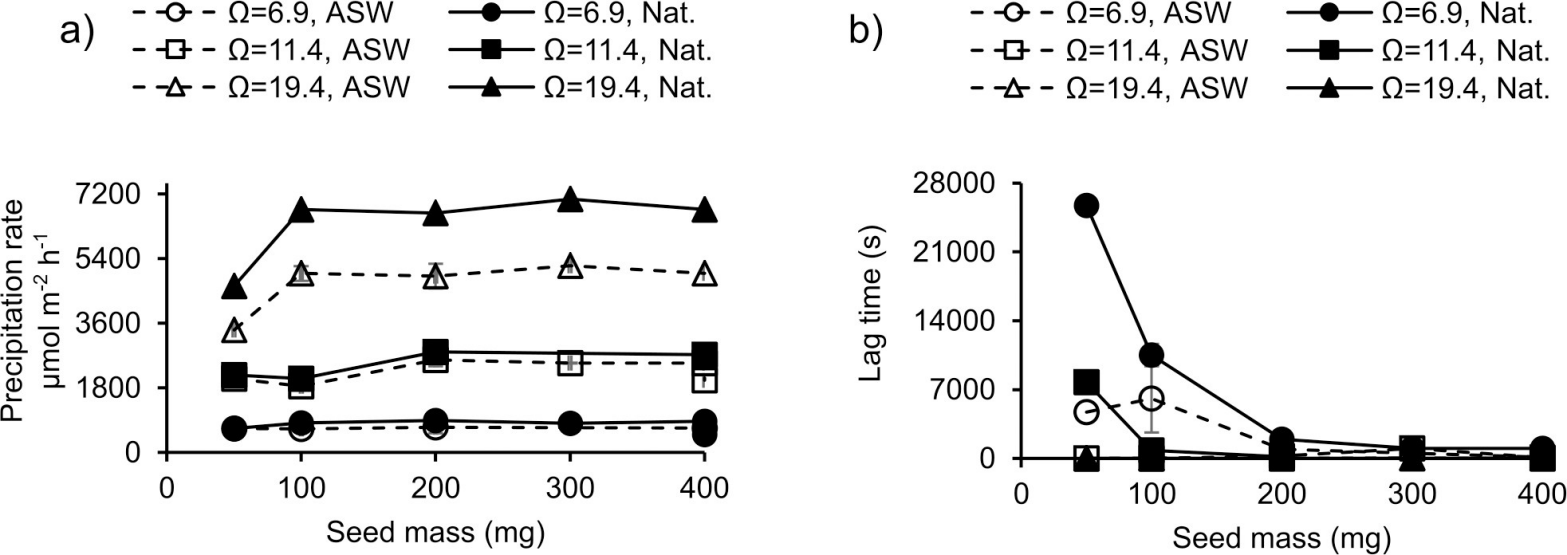
Estimated a) aragonite precipitation rate and b) lag period at start of precipitation in experiments comparing Ω, starting seed mass and different waters. ASW = artificial seawater. Nat. = natural seawater. Error bars indicate 1 σ of duplicate precipitations. The reproducibility of estimated precipitation rates from duplicate experiments was typically 6% and was always <10%. (Reproducibility of lag periods was always better than 57%).

Some profiles exhibited lag periods; these were more frequent during precipitations that ran at lower saturation states (Fig 4). To estimate lag periods and precipitation rates from the profiles we calculated predicted titration profiles for (as for Fig 5, using the known amount of added seed), we plotted a third order polynomial regression through the prediction profile and varied precipitation rate in the prediction to achieve the highest coefficient of determination (r^2^) between the predicted data and that observed in the precipitations [34]. For the precipitations in which a lag is observed we added a lag period to the prediction profile model. We identify the time at which 1 mL of each titrant is dosed on the experimental profiles. We subtract the time to dose 1 mL on the predicted profile from the experimental profile to determine the lag and we add this lag period to each time point on the predicted profile. In essence we are shifting the entire predicted profile to the right so that the points where 1 mL of titrant are dosed in the experimental and predicted profiles coincide. To estimate precipitation rate we again varied precipitation rate in the prediction profile to achieve the highest coefficient of determination (r^2^) between the observed and predicted data for the addition of 1–5 mL titrant. We overlay all predictions (with or without lag) onto Fig 5 (as red dotted lines) and, in the case of a lag, extend the profile to the y axis. We observe an excellent fit (r^2^ = 0.999) between the predicted and observed profiles in all precipitations with no lag (all precipitations at Ω = 19.2 and for those in artificial seawater at Ω = 11.4). The curve in the profile at low seed mass is reproduced in the predicted profiles suggesting that the curve reflects an increase in the surface area available for CaCO_3_ precipitation. Precipitation rates and lag periods for each set of conditions are summarised in Fig 6A and 6B respectively. Lag periods less than ~300 s could not be confidently identified as the addition of seed at the start of each experiment took 60–120 s and minor lags in the onset of dosing could reflect this delay.

Heterogeneous nucleation occurs in the presence of existing nucleation surfaces (e.g. a mineral seed), and involves the formation of a crystalline nucleus on the existing mineral surface and subsequent growth from that point. Lag periods reflect delays in this nucleation step and were most apparent at low Ω and/or seed mass and in the natural seawater (Fig 6). Lag periods >1800 s (20 minutes) occurred in both natural and artificial seawater at Ω = 6.9 with 50 and 100 mg seed and in natural seawater at Ω = 11.4 with 50 mg seed. Lag periods in the seeded experiments at Ω = 19.2 were insignificant and at Ω = 6.9 were much shorter than in unseeded experiments e.g. ~4000 s in artificial seawater with 50 mg seed compared to up to >100000 s in the unseeded analogue. This indicates that the seed provides an important surface for heterogeneous nucleation, even in the experiments with a lag period. Heterogeneous nucleation may occur on both the seed and the surface of the apparatus (beaker, sensor, titrant dosing tubes, stirrer). However the surface area of the seed (0.210 m^2^ for a 50 mg aliquot of seed) far exceeds that of the apparatus in contact with the seawater (estimated to be <0.03 m^2^, assuming the 73 mm diameter beaker is filled to a depth of 8.2 cm, the 12 mm diameter pH sensor is immersed to a depth of 6 cm, the 15 mm diameter (at widest point) stirrer is immersed to a depth of 7.5 cm and the 2 x 2 mm diameter titrant dosing tubes are immersed to a depth of 7.5 cm). The longer lag in natural seawater is discussed in section 3.3.4.

Precipitation rates normalised to seed surface area are comparable between duplicate experiments (typically 6%), and similar rates (i.e. within 6%) were estimated from experiments using 200, 300 and 400 mg seed. Precipitations normalised to 50 mg seed surface area yielded rates that were typically 70–80% of the rates observed at seed masses of 200–400 mg. Under only one set of conditions (Ω = 6.8, artificial seawater) did precipitation rates from 50 mg agree with those from higher seed masses within duplicate error. It’s not clear why precipitation rate is slower at small seed mass. We occasionally observed that some precipitate collected in a rim around the edge of the HDPE beaker during the experiment. This clumping may reflect an electrostatic attraction between the precipitate and the vessel and could reduce the CaCO_3_ surface area in contact with the seawater and available for precipitation. Clumping is likely to have a larger effect at low seed mass when experiments are longer in duration (with more time to clump) and when any clumping will have a proportionally larger effect (due to the low starting seed mass).

#### 3.3.2 Effect of seed on Raman signature of *in vitro* precipitates

**Fig 7:**
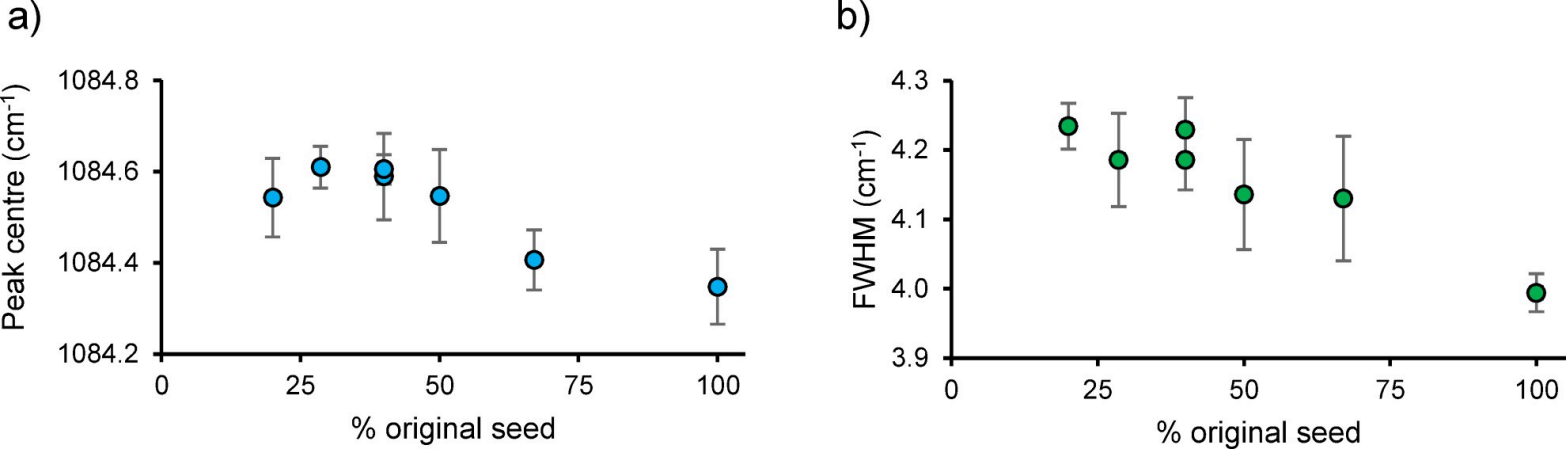
Peak centre and FWHM of the ν1 peak in the Raman spectra of precipitates containing different proportions of seed and *in vitro* precipitate. a) Peak centre and b) Full width half maxima. The signatures of the seed are shown by the point at 100% seed. Points are means of 10 spectra and error bars are 1 standard deviation.

We analysed the aragonite *v*_1_ peak centre and FWHM of precipitates containing varying proportions of seed and *in vitro* precipitate (Fig 7). The peak centres and FWHM of all *in vitro* precipitates were significantly higher than that of the seed alone (ANOVA, p ≤ 0.05). We observed no significant differences in the peak centre or FWHM between *in vitro* precipitates containing ≤40% of the seed by mass. Our data suggests that the Raman signature of the *in vitro* precipitate can be resolved successfully if at least 60% of the analysed sample is precipitated *in vitro*.

#### 3.3.3 Optimising the aragonite precipitation method

In this study we optimise a method for the precipitation of aragonite *in vitro* and normalise to the influences of seed mass and experiment duration on the final precipitate. Experiments with no seed are lengthy and any disequilibria between the atmospheric and seawater CO_2_ can result in CO_2_ invasion or outgas from the seawater solution creating a drift in both the [DIC] and Ω of the seawater solution. In this study unseeded experiments replicate poorly and exhibit different precipitation rates which may be an important control on aragonite geochemistry and structure. We consider unseeded experiments unsuitable for investigating the effects of environment and precipitation rate on geochemistry and structure. Adding a very small surface area/mass of seed can result in slow experiments which yield low precipitation rates (as observed in 50 mg seeded experiments above). Adding a larger surface area/mass of seed accelerates the experiment and we observed good agreement in precipitation rate estimates from experiments using 200–400 mg seed. Invasion and outgas of CO_2_ is minor in these shorter experiments and measured DIC at the start and end of the precipitations using 200 mg seed agreed with predicted [DIC] within 2% at pH_NBS_ = 8.445 and 8.564, and within 3% at pH_NBS_ = 8.337. However, increasing the amount of seed also increases the contamination of the geochemistry and structure of the final precipitate (seed plus *in vitro* precipitate) by the starting seed. Our analyses indicates that the Raman signature of the *in vitro* precipitate can be distinguished from the seed when the analysed sample is ≤40% of the seed by mass. In our experimental design this is equivalent to using a seed of 200 mg and precipitating 300 mg of aragonite *in vitro*. We have adopted this as our optimised methodology. Precipitating >300 mg *in vitro* will further reduce the domination of the precipitate by the seed, but it is likely that precipitating larger masses of aragonite has implications for the trace and minor element and isotopic compositions of the seawater solution used for the process of precipitation itself. It is all but impossible to produce titrant solutions that exactly replace the ions consumed during precipitation, particularly as element/isotope partitioning is likely to vary under different experimental conditions (e.g. pH, Ω). For example, seawater Mg/Ca varied by 1–7% during precipitation of amorphous calcium carbonate from seawater under different pH, DIC chemistry and biomolecule availability [35]. The more aragonite precipitated from a solution, the larger the changes in the seawater composition will be, and this in itself will influence the partitioning of minor and trace elements and isotopes into aragonite.

#### 3.3.4 Effect of aspartic acid on aragonite precipitation rate and structure

**Fig 8:**
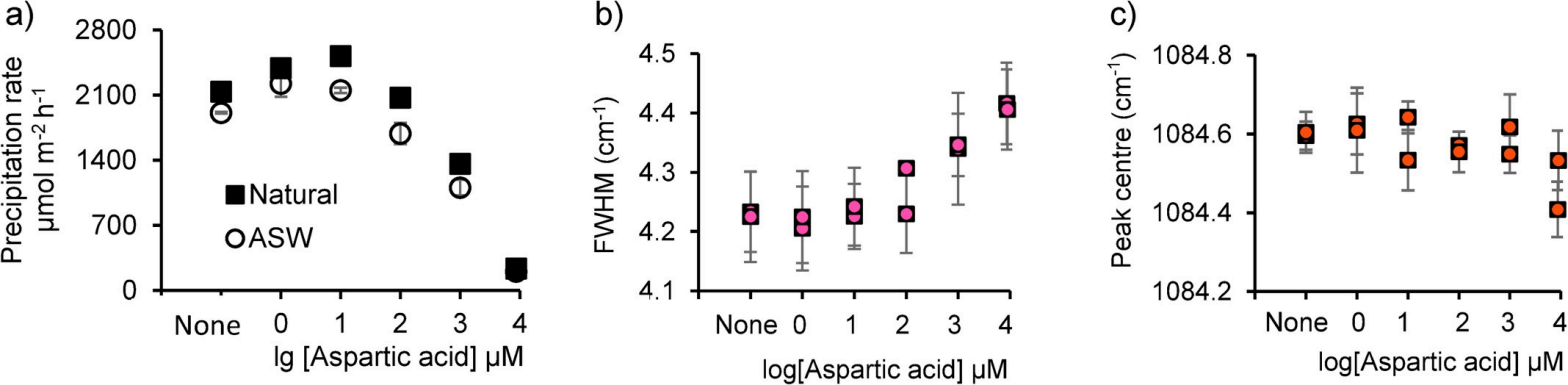
a) Aragonite precipitation rates from natural and artificial seawater in the presence of a range of concentrations of aspartic acid at Ω = 11.2. Precipitations were conducted in duplicate; points indicate mean rate and error bars indicate 1 standard deviation. The error bars are usually smaller than the symbols. b) FWHM and c) peak position of the *v*_1_ peak in the Raman spectra of aragonites precipitated in the presence of aspartic acid. Points are means of 10–11 spectra and error bars are 1 standard deviation.

Aragonite precipitation rates were accelerated significantly by the addition of low concentrations of aspartic acid (by 1 and 10 μM in artificial and natural seawaters respectively) and inhibited by concentrations of ≥1 mM in both waters (ANOVA, p ≤ 0.05, Fig 8). Low concentrations of biomineralisation proteins [36], aspartic acid [37] and multiple residue aspartic acid peptides (aspartates) [38] have previously been found to promote the propagation of calcite crystals, while higher concentrations can inhibit propagation compared to controls [38]. Biomolecules play significant roles in controlling CaCO_3_ precipitation and may operate by decreasing the energy barrier to ion attachment at the growing crystal face [38], or by blocking ion attachment [39], or by binding the dissolved Ca^2+^ required for CaCO_3_ precipitation [40]. Exquisite control over aragonite precipitation is required to produce the highly organised, regular skeletons deposited by corals [41]. Aspartic acid is the predominant amino acid in Scleractinia coral skeletons [17, 18] and is likely involved in the control of coral biomineralisation as the carboxyl acid side chain of this amino acid is negatively charged at physiological pH and may electrostatically attract Ca^2+^ at the crystal surface [42]. Comparing the concentrations of aspartic acid incorporated in synthetic aragonite with those of coral skeletons suggests the [aspartic acid] of the coral calcification media is ~100–400 μM [18]. At these concentrations it is unclear if the amino acid acts to promote or inhibit aragonite growth (Fig 8). Aspartic acid predominantly occurs in coral skeletons as peptides and proteins, so further research is required to determine how these larger molecules influence aragonite precipitation. Low concentrations (0.1 μM) of large aspartic-rich peptides enhance calcite growth by a much greater magnitude than smaller peptides (of 6 aspartic acid residue or less) [38] but there is no current estimate of the likely concentrations of proteins at the coral calcification site to establish if these molecules truly promote or inhibit coral aragonite formation.

The concentrations of organic materials, [43, 44] aspartic acid in particular, [18] increase in the skeletons of corals cultured under high seawater pCO_2_ (ocean acidification). If skeletal organics act to promote aragonite precipitation, then these increases could reflect a coral response to compensate for the reduced omega of seawater under ocean acidification, which typically inhibits coral calcification [44]. If skeletal organics inhibit aragonite precipitation, then this response to high seawater pCO_2_ acts to intensify the reduction in coral calcification under ocean acidification [18].

Aragonite precipitation rates were significantly faster in natural compared to artificial seawater (paired t test, p = 0.0069), typically by ~19%. Although filtered before use, natural seawater contains a mixture of dissolved organic matter (DOM) not present in the artificial seawater which may influence CaCO_3_ precipitation. Marine DOM is a complex mixture of biomolecules derived from marine and terrestrial sources (e.g. phytoplankton metabolism and plant decay products) and is typically 25–50% protein, 2–25% lipid and up to 40% carbohydrate [45]. Most seawater DOM has a molecular weight of <1 kDa (i.e. <1000 g per mole) as larger mass DOM is more readily biodegraded [46]. The majority of amino acids in the DOM occur as combined amino acids (i.e. as low molecular weight peptides, rather than free amino acids) [47]. Proteins smaller than 1 kDa contain ~10 amino acid residues or less and can be considered as peptides. Aspartic acid is one of the main constituents of the free and combined amino acids in seawater DOM [48] and can exceed concentrations of 1 μM [47]. In our study, [aspartic acid] of 1 μM promotes aragonite formation in artificial seawater and the likely inclusion of low levels of DOM in the natural seawater may explain why aragonite precipitation rates are typically higher in these waters. For this study the natural seawater was stored in the dark for several weeks before use. In the absence of phototropic organisms it is likely that the DOM will decrease as it is utilised by heterotrophic bacteria [47]. Although total dissolved amino acid likely decreased during storage, the contribution of seawater aspartic acid to the total amino acid likely increased, reflecting the discrimination against this amino acid during microbial heterotrophy [47]. We note that precipitation lag times (when they occur) are longer in natural compared to artificial seawater (Fig 6). High concentrations of aspartic acid (10 mM) can delay CaCO_3_ nucleation and increase the solubility of initial CaCO_3_ phases [49] but it is unclear if these effects are induced by DOM in seawater. The complexation of Ca^2+^_(aq)_ by DOM may also impact heterogenous nucleation.

**Fig 9:**
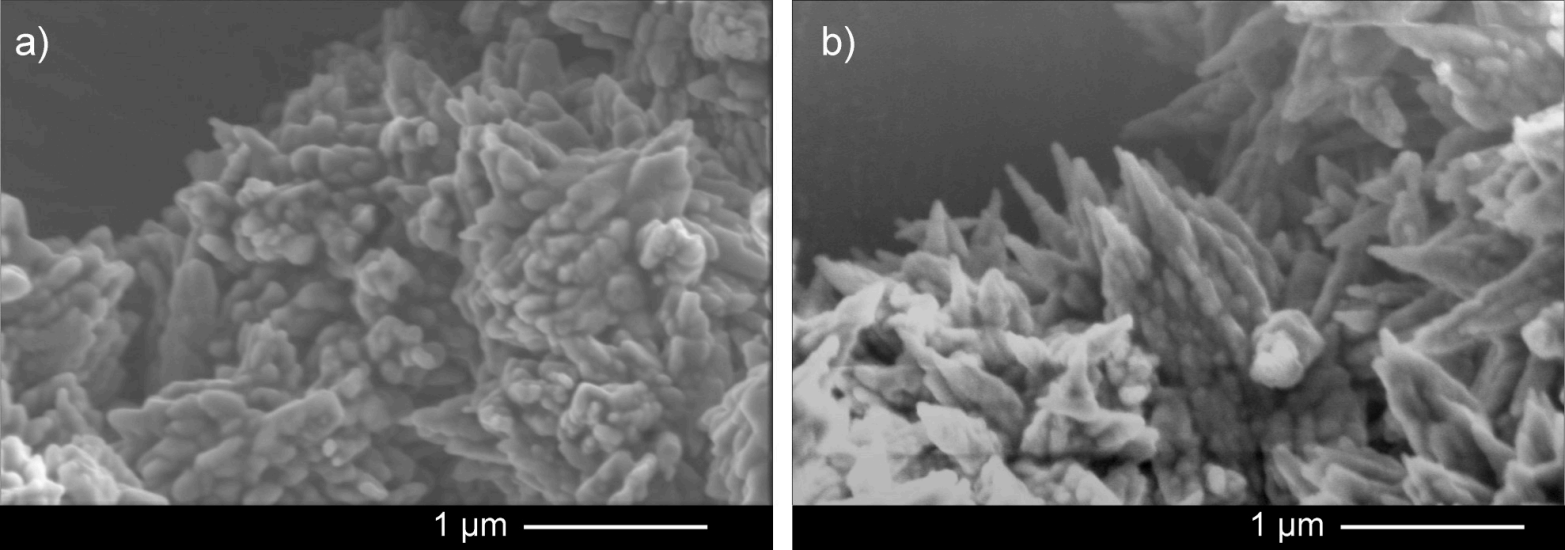
Scanning electron micrographs of aragonite precipitated at Ω = 11.2. a) without aspartic acid and b) with 8.7 mM aspartic acid. Scale bars are 1 μm.

Aragonites precipitated in the presence of aspartic acid were composed of larger, more pointer crystals than their counterparts precipitated in artificial seawater with no biomolecules (Fig 9). This suggests that the slower aragonite precipitation rates observed at higher concentrations of aspartic acid resulted in the formation of larger crystals.

Low concentrations of aspartic acid (1 and 10 μM) did not significantly affect the ν_1_ aragonite peak in the Raman spectra, but higher concentrations (≥1 mM) caused an increase in FWHM of these bands compared to the aragonite precipitated with no aspartic acid (Fig 8, ANOVA, p<0.05). Aspartic acid additions did not affect the peak position. The ν_1_ band results from symmetric C-O stretching in the planar carbonate ion [50] and broadening of this peak is indicative of increased local disorder around the CO_3_^2-^ ion. We considered if broadening of the ν_1_ peak could reflect incorporation of another CaCO_3_ phase e.g. calcite or amorphous calcium carbonate in the precipitated aragonite. Representative Raman spectra of all precipitates are included in the S1 Data. All spectra exhibit a pronounced dual peak at 700–710 cm^-1^, indicative of aragonite [30]. Furthermore, the lattice mode vibrations observed at 100–250 cm^-1^ in all precipitates are also consistent with aragonite with no evidence of the features associated with calcite or amorphous calcium carbonate [51]. Finally, we note that we have been unable to precipitate amorphous calcium carbonate in our laboratory at the pH and Ω tested here, even in the presence of aspartic acid [52]. Collectively, we find no evidence that non-aragonitic CaCO3 phases contribute to ν_1_ peak broadening. The primary cause of the loss of short-range order in the precipitates remains unclear. Rotational disorder can be caused by rapid disequilibrium crystal growth [4] or can be a consequence of the incorporation of contaminant ions in the crystal lattice which create local lattice distortions [3].

Whatever its cause, the Raman aragonite ν_1_ peak FWHM has been linked to increases in the Ω of the seawater solution used for precipitation [4] and to the inclusion of biomolecules [53]. Similarly, in coral skeletons, broadening of the ν1 peak is observed in the centres of calcification (the features at the centres of the skeletal units) and may reflect increased calcification media omega [54] or increased skeletal organic material [55]. The FWHM of the aragonite ν1 peak has previously been used to infer the saturation state of the coral calcification fluid [4] and to track the calcification response of corals to increased seawater pCO_2_. However, our study demonstrates that the inclusion of biomolecules in aragonite can influence aragonite disorder even at constant fluid saturation state. Therefore, the increases in the organic and amino acid contents of skeletons of corals cultured at high seawater pCO_2_ [18, 43, 44] are likely to influence the structural disorder of the aragonite skeletons and potentially obscure any calcification fluid/seawater Ω signal. Further work is required to clarify how the inclusion of aspartic acid in peptides and proteins (as in biogenic aragonites) affects the aragonite precipitation rate and structure.

## 4. Conclusions

We optimised a method for the precipitation of synthetic aragonites under simulated biological conditions by using aragonite seed as a substrate to improve reproducibility of precipitation rate in comparison with unseeded experiments. Initial application of this method shows that aspartic acid, the most common amino acid in Scleratinian coral skeletons, promotes aragonite precipitation at low concentrations (1 and 10 μM) but inhibits precipitation at concentrations ≥1mM. Aragonite crystals precipitated in the presence of high concentrations of aspartic acid have wider FWHM of the Raman spectrum v_1_ peak (indicative of the carbonate ion symmetric stretch) suggesting that the biomolecule disrupts the rotational structural order of the aragonite lattice. Future changes in seawater chemistry (influenced by climate) may alter the current ratios of organic molecules in seawater and therefore alter the contributions of aspartic acid (and other amino acids) to coral skeletons, highlighting the relevance and importance of being able to simulate precipitation conditions and scenarios. A standard and reliable precipitation method is necessary to further investigate the influence of changing environmental conditions on the geochemistry of coral skeletons, and to produce comparable results and advance research in this field.

## Supporting information

S1 Fig Raman of aspartic acid. (DOCX)

S1 Data This file consists of the supporting data tables. (XLSX)

## References

[1] Beck JW, Edwards RL, Ito E, Taylor FW, Recy J, Rougerie F, et al. Sea-surface temperature from coral skeletal strontium/calcium ratios. Science. 1992 Jul 31;257(5070):644–7. doi: 10.1126/science.257.5070.644 17740731

[2] Nurnberg D, Bijma J, Hemleben C. Assessing the reliability of magnesium in foraminiferal calcite as a proxy for water mass temperatures. Geochimica et cosmochimica Acta. 1996 Mar 1;60(5):803–14.

[3] Kamenos NA, Burdett HL, Aloisio E, Findlay HS, Martin S, Longbone C, et al. Coralline algal structure is more sensitive to rate, rather than the magnitude, of ocean acidification. Global Change Biology. 2013 Dec;19(12):3621–8. doi: 10.1111/gcb.12351 23943376 PMC4285748

[4] De Carlo TM D’Olivo JP, Foster T, Holcomb M, Becker T, McCulloch MT. Coral calcifying fluid aragonite saturation states derived from Raman spectroscopy. Biogeosciences. 2017 Nov 24;14(22):5253–69.

[5] Friedrich T, Timmermann A, Tigchelaar M, Elison Timm O, Ganopolski A. Nonlinear climate sensitivity and its implications for future greenhouse warming. Science Advances. 2016 Nov 9;2(11):e1501923. doi: 10.1126/sciadv.1501923 28861462 PMC5569956

[6] Gaetani GA, Cohen AL. Element partitioning during precipitation of aragonite from seawater: a framework for understanding paleoproxies. Geochimica et cosmochimica acta. 2006 Sep 15;70(18):4617–34.

[7] Dietzel M, Gussone N, Eisenhauer A. Co-precipitation of Sr2+ and Ba2+ with aragonite by membrane diffusion of CO2 between 10 and 50 C. Chemical Geology. 2004 Jan 15;203(1–2):139–51.

[8] Burton EA, Walter LM. The effects of PCO2 and temperature on magnesium incorporation in calcite in seawater and MgCl2-CaCl2 solutions. Geochimica et Cosmochimica Acta. 1991 Mar 1;55(3):777–85.

[9] Holcomb M et al. Factors affecting B/Ca ratios in synthetic aragonite. Chem. Geol. 437, 67–76, 2016.

[10] Mavromatis V, Gautier Q, Bosc O, Schott J. Kinetics of Mg partition and Mg stable isotope fractionation during its incorporation in calcite. Geochimica et Cosmochimica Acta. 2013 Aug 1;114:188–203.

[11] Mavromatis V, Immenhauser A, Buhl D, Purgstaller B, Baldermann A, Dietzel M. Effect of organic ligands on Mg partitioning and Mg isotope fractionation during low-temperature precipitation of calcite in the absence of growth rate effects. Geochimica et Cosmochimica Acta. 2017 Jun 15;207:139–53.

[12] Gabitov RI, Sadekov A, Leinweber A. Crystal growth rate effect on Mg/Ca and Sr/Ca partitioning between calcite and fluid: An in situ approach. Chemical Geology. 2014 Feb 28;367:70–82.

[13] Watson EB. A conceptual model for near-surface kinetic controls on the trace-element and stable isotope composition of abiogenic calcite crystals. Geochimica et cosmochimica acta. 2004 Apr 1;68(7):1473–88.

[14] AlKhatib M, Eisenhauer A. Calcium and strontium isotope fractionation during precipitation from aqueous solutions as a function of temperature and reaction rate; II. Aragonite. Geochimica et Cosmochimica Acta. 2017 Jul 15;209:320–42.

[15] Burton EA, Walter LM. Relative precipitation rates of aragonite and Mg calcite from seawater: Temperature or carbonate ion control?. Geology. 1987 Feb 1;15(2):111–4.

[16] Nehrke G, Nouet J. Confocal Raman microscope mapping as a tool to describe different mineral and organic phases at high spatial resolution within marine biogenic carbonates: case study on Nerita undata (Gastropoda, Neritopsina). Biogeosciences. 2011 Dec 20;8(12):3761–9.

[17] Cuif JP, Dauphin Y, Freiwald A, Gautret P, Zibrowius H. Biochemical markers of zooxanthellae symbiosis in soluble matrices of skeleton of 24 Scleractinia species. Comparative Biochemistry and Physiology Part A: Molecular & Integrative Physiology. 1999 Jul 1;123(3):269–78.

[18] Kellock C, Cole C, Penkman K, Evans D, Kröger R, Hintz C, et al. The role of aspartic acid in reducing coral calcification under ocean acidification conditions. Scientific reports. 2020 Jul 30;10(1):1–8. 32733044 10.1038/s41598-020-69556-0 PMC7393068

[19] Puverel S, Tambutté E, Pereira-Mouriès L, Zoccola D, Allemand D, Tambutté S. Soluble organic matrix of two Scleractinian corals: partial and comparative analysis. Comparative Biochemistry and Physiology Part B: Biochemistry and Molecular Biology. 2005 Aug 1;141(4):480–7. doi: 10.1016/j.cbpc.2005.05.013 15982916

[20] Finch AA, Allison N, Sutton SR, Newville M. Strontium in coral aragonite: 1. Characterization of Sr coordination by extended absorption X-ray fine structure. Geochimica et Cosmochimica Acta. 2003 Mar 15;67(6):1197–202.

[21] Millero FJ, Chemical Oceanography, 4th ed.; CRC Press: Boca, Raton, FL, 2013.

[22] Cole C, Finch A, Hintz C, Hintz K, Allison N. Understanding cold bias: variable response of skeletal Sr/Ca to seawater pCO2 in acclimated massive Porites corals. Scientific reports. 2016 May 31;6(1):1–8. 27241795 10.1038/srep26888 PMC4886260

[23] Mucci A. The solubility of calcite and aragonite in seawater at various salinities, temperatures, and one atmosphere total pressure. Am. J. Sci. 1983 Sep 1;283(7):780–99.

[24] Pierrot D, Lewis ED, Wallace WR, 2006 MS Excel Program Developed for CO2 System Calculations. Oak Ridge National Laboratory.

[25] Lueker TJ, Dickson AG, Keeling CD. Ocean pCO2 calculated from dissolved inorganic carbon, alkalinity, and equations for K1 and K2: validation based on laboratory measurements of CO2 in gas and seawater at equilibrium. Marine chemistry. 2000 May 1;70(1–3):105–19.

[26] Dickson AG. Standard potential of the reaction: AgCl (s)+ 12H2 (g) = Ag (s)+ HCl (aq), and and the standard acidity constant of the ion HSO4− in synthetic sea water from 273.15 to 318.15 K. The Journal of Chemical Thermodynamics. 1990 Feb 1;22(2):113–27.

[27] Uppström LR. The boron/chlorinity ratio of deep-sea water from the Pacific Ocean. Deep Sea Res. 1974;21:161–2.

[28] Sevilgen DS, Venn AA, Hu MY, Tambutté E, de Beer D, Planas-Bielsa V, et al. Full in vivo characterization of carbonate chemistry at the site of calcification in corals. Science advances. 2019 Jan 16;5(1):eaau7447. doi: 10.1126/sciadv.aau7447 30746460 PMC6357752

[29] Meier RJ. On art and science in curve-fitting vibrational spectra. Vibrational spectroscopy. 2005;2(39):266–9.

[30] Urmos J, Sharma SK, Mackenzie FT. Characterization of some biogenic carbonates with Raman spectroscopy. American Mineralogist. 1991 Apr 1;76(3–4):641–6.

[31] Gebauer D, Raiteri P, Gale JD, Cölfen H. On classical and non-classical views on nucleation. American Journal of Science. 2018 Nov 1;318(9):969–88.

[32] Morse JW, He S. Influences of T, S and PCO2 on the pseudo-homogeneous precipitation of CaCO3 from seawater: implications for whiting formation. Marine Chemistry. 1993 Feb 1;41(4):291–7.

[33] Morse JW, Gledhill DK, Millero FJ. Caco3 precipitation kinetics in waters from the great Bahama bank:: Implications for the relationship between bank hydrochemistry and whitings. Geochimica et Cosmochimica Acta. 2003 Aug 1;67(15):2819–26.

[34] Ostertagová E. Modelling using polynomial regression. Procedia Engineering. 2012 Jan 1;48:500–6.

[35] Evans D, Gray WR, Rae JW, Greenop R, Webb PB, Penkman K, et al. Trace and major element incorporation into amorphous calcium carbonate (ACC) precipitated from seawater. Geochimica et Cosmochimica Acta. 2020 Dec 1;290:293–311.

[36] Fu G, Qiu SR, Orme CA, Morse DE, De Yoreo JJ. Acceleration of calcite kinetics by abalone nacre proteins. Advanced materials. 2005 Nov 18;17(22):2678–83.

[37] Elhadj S, Salter EA, Wierzbicki A, De Yoreo JJ, Han N, Dove PM. Peptide controls on calcite mineralization: Polyaspartate chain length affects growth kinetics and acts as a stereochemical switch on morphology. Crystal growth & design. 2006 Jan 4;6(1):197–201.

[38] Elhadj S, De Yoreo JJ, Hoyer JR, Dove PM. Role of molecular charge and hydrophilicity in regulating the kinetics of crystal growth. Proceedings of the National Academy of Sciences. 2006 Dec 19;103(51):19237–42. doi: 10.1073/pnas.0605748103 17158220 PMC1748210

[39] Sikirić MD, Füredi-Milhofer H. The influence of surface active molecules on the crystallization of biominerals in solution. Advances in colloid and interface science. 2006 Dec 21;128:135–58. doi: 10.1016/j.cis.2006.11.022 17254533

[40] Tong H, Ma W, Wang L, Wan P, Hu J, Cao L. Control over the crystal phase, shape, size and aggregation of calcium carbonate via a L-aspartic acid inducing process. Biomaterials. 2004 Aug 1;25(17):3923–9. doi: 10.1016/j.biomaterials.2003.10.038 15020169

[41] Drake JL, Mass T, Stolarski J, Von Euw S, van de Schootbrugge B, Falkowski PG. How corals made rocks through the ages. Global change biology. 2020 Jan;26(1):31–53. doi: 10.1111/gcb.14912 31696576 PMC6942544

[42] Wierzbicki A, Sikes CS, Madura JD, Drake B. Atomic force microscopy and molecular modeling of protein and peptide binding to calcite. Calcified tissue international. 1994 Feb;54(2):133–41. doi: 10.1007/BF00296064 8012868

[43] Coronado I, Fine M, Bosellini FR, Stolarski J. Impact of ocean acidification on crystallographic vital effect of the coral skeleton. Nature communications. 2019 Jul 1;10(1):1–9. 10.1038/s41467-019-10833-6 PMC6603003 31263108

[44] Tambutté E, Venn AA, Holcomb M, Segonds N, Techer N, Zoccola D, et al. Morphological plasticity of the coral skeleton under CO2-driven seawater acidification. Nature Communications. 2015 Jun 12;6(1):1–9. doi: 10.1038/ncomms8368 26067341 PMC4490415

[45] Nebbioso A, Piccolo A. Molecular characterization of dissolved organic matter (DOM): a critical review. Analytical and bioanalytical chemistry. 2013 Jan;405(1):109–24. 22965531 10.1007/s00216-012-6363-2

[46] Penru Y, Simon FX, Guastalli AR, Esplugas S, Llorens J, Baig S. Characterization of natural organic matter from Mediterranean coastal seawater. Journal of Water Supply: Research and Technology—AQUA. 2013 Feb;62(1):42–51.

[47] Sommerville K, Preston T. Characterisation of dissolved combined amino acids in marine waters. Rapid Communications in Mass Spectrometry. 2001 Aug 15;15(15):1287–90. doi: 10.1002/rcm.302 11466786

[48] Ji CX, Yang GP, Chen Y, Zhang PY. Distribution, degradation and bioavailability of dissolved organic matter in the East China Sea. Biogeochemistry. 2019 Jan;142(2):189–207.

[49] Picker A, Kellermeier M, Seto J, Gebauer D, Cölfen H. The multiple effects of amino acids on the early stages of calcium carbonate crystallization. Zeitschrift für Kristallographie-Crystalline Materials. 2012 Nov 1;227(11):744–57.

[50] Bischoff WD, Sharma SK, MacKenzie FT. Carbonate ion disorder in synthetic and biogenic magnesian calcites: a Raman spectral study. American Mineralogist. 1985 Jun 1;70(5–6):581–9.

[51] DeCarlo TM. Characterizing coral skeleton mineralogy with Raman spectroscopy. Nature Communications. 2018 Dec 14;9(1):1–3. 10.1038/s41467-018-07601-3 PMC6293996 30552319

[52] Evans D, Webb PB, Penkman K, Kroger R, Allison N. The characteristics and biological relevance of inorganic amorphous calcium carbonate (ACC) precipitated from seawater. Crystal Growth & Design. 2019 Jul 3;19(8):4300–13.

[53] Ihli J, Clark JN, Kanwal N, Kim YY, Holden MA, Harder RJ, et al. Visualization of the effect of additives on the nanostructures of individual bio-inspired calcite crystals. Chemical science. 2019;10(4):1176–85. doi: 10.1039/c8sc03733g 30774916 PMC6349071

[54] Sharma SK, Urmos JP. Micro-Raman spectroscopic studies of materials at ambient and high pressures with CW and pulsed lasers. Microbeam Analysis. 1987:133–6.

[55] Von Euw S, Zhang Q, Manichev V, Murali N, Gross J, Feldman LC, et al. Biological control of aragonite formation in stony corals. Science. 2017 Jun 2;356(6341):933–8. doi: 10.1126/science.aam6371 28572387

