# Supplementary material for: Optimising a method for aragonite precipitation in simulated biogenic calcification media
Source: PLoS One. 2022 Dec 2;17(12):e0278627. doi: 10.1371/journal.pone.0278627 (PMC9718392; doi:10.1371/journal.pone.0278627)
Supplement: S1 Fig — (DOCX) [file pone.0278627.s001.docx]

**Supplementary Data**

**PONE-D-22-18432**

**Optimising a method for aragonite precipitation in simulated biogenic calcification media**

Celeste Kellock^1a*^, Maria Cristina Castillo Alvarez^1^, Adrian Finch^1^, Kirsty Penkman^2^, Roland Kröger^3^, Matthieu Clog^4^ & Nicola Allison^1^

**Corresponding author, email: cek3@stir.ac.uk*

**Supplementary Figure S1: Raman spectra of aspartic acid.** Orange line shows the aragonite profile, black line shows the aspartic acid profile.
